# Prevalence of mammary Paget’s disease in urban China in 2016

**DOI:** 10.1038/s41598-021-82146-y

**Published:** 2021-01-28

**Authors:** Lu Xu, Shilu Yin, Shengfeng Wang, Jingnan Feng, Lili Liu, Guozhen Liu, Jinxi Wang, Siyan Zhan, Zhenmin Zhao, Pei Gao

**Affiliations:** 1 Department of Epidemiology and Biostatistics, School of Public Health, Peking University, 38 Xueyuan Road, Haidian District, Beijing, 100191 China; 2 Department of Plastic Surgery, Peking University Third Hospital, 49 North Garden Road, Haidian District, Beijing, 100191 China; 3 Peking University Health Information Technology Co. Ltd, 52 North Fourth Ring West Road, Haidian District, Beijing, 100080 China; 4 Shanghai Songsheng Business Consulting Co. Ltd, 6 Chaoyang Men North Street, Dongcheng District, Beijing, 100000 China; 5 Research Center of Clinical Epidemiology, Peking University Third Hospital, 49 North Garden Road, Haidian District, Beijing, 100191 China; 6 Center for Intelligent Public Health, Institute for Artificial Intelligence, Peking University, 38 Xueyuan Road, Haidian District, Beijing, 100191 China

**Keywords:** Cancer, Breast cancer, Cancer epidemiology

## Abstract

No national data have been available on descriptive epidemiology of mammary Paget’s disease (MPD) in China. This population-based study aimed to estimate the prevalence of MPD and its pattens by sex, age and area in China. We conducted a population-based study using data in 2016 from China’s Urban Employee Basic Medical Insurance and Urban Resident Basic Medical Insurance, covering approximately 0.43 billion residents. MPD cases were identified based on the diagnostic names and codes in claim data. A total of 825 patients of confirmed diagnosis of MPD were found during the study period. The prevalence of MPD in 2016 was 0.42 per 100,000 population (95% CI 0.19 to 0.73), with marked female predominance. The prevalence rates peaked at 40–59 years and ≥ 80 years in females and males, respectively. The prevalence rates varied among different regions, ranging from 0.06 (95% CI 0.00 to 0.23) in Northeast China to 1.21 (95% CI 0.07 to 3.72) in Northwest China. MPD showed marked female predominance in China. Chinese female patients were much younger, with lower prevalence than that in the United States. Obvious sex difference in the age pattern of MPD prevalence was also observed in China.

## Introduction

Mammary Paget’s disease (MPD) is an uncommon clinical entity characterized by eczematous changes and ulceration, which usually affect the nipple or areola^1,2^. It has different clinical presentations from other pathologic subtypes of breast cancer^3,4^. Symptoms of MPD such as itching, skin ulcer and bleeding, often persist over a long period of time and lead to a decrease of quality of life^5^. However, epidemiological information of MPD was limited by the paucity of published data, especially in the developing countries including China.

MPD was reported to account for 0.7–4.3% of all breast cancers^2,6^, and approximately 82–100% of cases were associated with underlying in situ or invasive breast cancer^2,6,7^. In China, female patients with MPD was reported to account for 1.6% of all female patients with primary breast cancers in multi-center hospital-based study during 1999–2008^8^. The incidences of MPD were reported in two studies based on analysis of the Surveillance, Epidemiology, and End Results (SEER) database in the United States, which ranged from 0.44 per 100,000 woman-years to 1.31 per 100,000 woman-years between 1988 and 2011^9^. In western literatures, MPD is reported to be most commonly occurred in postmenopausal women during the sixth decade of life, but it has also been reported in adolescents (≥ 13 years) and oldest-old patients (≥ 90 years)^5,7,10,11^. By contrast, in Asian countries including Iran, India, South Korea, Turkey, Saudi Arabia and China, the female patients with MPD were much younger, with reported mean age mostly under 60 years old^3,8,12–19^. Men can also be affected, which accounts for 1.45% of all male breast cancers^20^, and the clinical characteristics are similar to those occurring in women^21^. To date, no epidemiological studies have been available to estimate the prevalence of MPD in mainland China.

This study was conducted to provide recent estimates of the prevalence of MPD in mainland China and to investigate its patterns across sexes, age groups and geographical areas, using a nationally representative sample in 2016.

## Results

Approximately 0.43 billion individuals were included in this study (Table 1). A total of 825 patients with MPD were observed in the database (Table 2). The mean age of the observed patients with MPD was 55.63 (SD = 12.17) years.

**Table 1:** Characteristics of the urban population from 23 provinces in China in 2016.

Characteristic	Total	Male	Female
Total number (million)	425.83	221.86	203.97
*Age (years)*			
Mean (SD)	37.68 (20.00)	37.21 (14.31)	38.20 (13.96)
*Age groups, n (%, million)*			
0–17	68.3 (16.04)	36.8 (16.59)	31.5 (15.44)
18–39	167.2 (39.26)	87.7 (39.53)	79.48 (38.97)
40–59	123.1 (28.91)	63.79 (28.75)	59.31 (29.08)
60–69	37.89 (8.90)	19.42 (8.75)	18.47 (9.05)
70–79	19 (4.46)	9.51 (4.29)	9.48 (4.65)
≥ 80	10.35 (2.43)	4.63 (2.09)	5.72 (2.80)
*Area, n (%, million)*			
East	171.30 (40.24)	88.68 (39.97)	82.67 (40.53)
North	18.92 (4.44)	9.63 (4.34)	9.28 (4.55)
Northeast	42.87 (10.07)	21.51 (9.69)	21.36 (10.47)
Northwest	21.60 (5.07)	11.25 (5.07)	10.35 (5.07)
Southcentral	123.60 (29.02)	66.18 (29.83)	57.41 (28.15)
Southwest	47.50 (11.15)	24.61 (11.09)	22.89 (11.22)

SD, standard deviation. Note: East area contains Jiangsu, Zhejiang, Anhui, Jiangxi, and Shandong (five provinces). North area contains Shanxi, Inner Mongolia (two provinces). Northeast contains Liaoning, Jilin, Heilongjiang (three provinces). Northwest contains Shaanxi, Qinghai, Gansu, and Xinjiang (four provinces). Southcentral contains Henan, Hubei, Hunan, Guangdong, Guangxi and Hainan (six provinces). Southwest contains Chongqing, Guizhou and Yunnan (three provinces).

**Table 2:** Characteristics of patients with mammary Paget's disease in Chinese 23 provinces in 2016.

Characteristic	Total	Male	Female	*P* value
Number	825	79	745	
*Age, years*				
Mean (SD)	55.63 (12.17)	68.80 (14.09)	54.23 (11.08)	< 0.001
*Age group, n (%)*				
0–17	1 (0.12)	0 (0)	1 (0.13)	< 0.001
18–39	61 (7.40)	5 (6.33)	56 (7.52)	
40–59	478 (58.01)	11 (13.92)	467 (62.68)	
60–69	172 (20.87)	14 (17.72)	158 (21.21)	
70–79	84 (10.19)	32 (40.51)	52 (6.98)	
≥ 80	28 (3.40)	17 (21.52)	11 (1.48)	
*Area, n (%)*				
East	399 (48.36)	28 (35.44)	371 (49.80)	< 0.001
North	42 (5.09)	0 (0)	42 (5.64)	
Northeast	32 (3.88)	1 (1.27)	31 (4.16)	
Northwest	136 (16.48)	23 (29.11)	113 (15.17)	
Southcentral	193 (23.39)	20 (25.32)	173 (23.22)	
Southwest	23 (2.79)	7 (8.86)	15 (2.01)	

SD, standard deviation. Note: a total of one patient had missing information in sex and age. East area contains Jiangsu, Zhejiang, Anhui, Jiangxi, and Shandong (five provinces). North area contains Shanxi, Inner Mongolia (two provinces). Northeast contains Liaoning, Jilin, Heilongjiang (three provinces). Northwest contains Shaanxi, Qinghai, Gansu, and Xinjiang (four provinces). Southcentral contains Henan, Hubei, Hunan, Guangdong, Guangxi and Hainan (six provinces). Southwest contains Chongqing, Guizhou and Yunnan (three provinces).

### Prevalence

**Figure 1:**
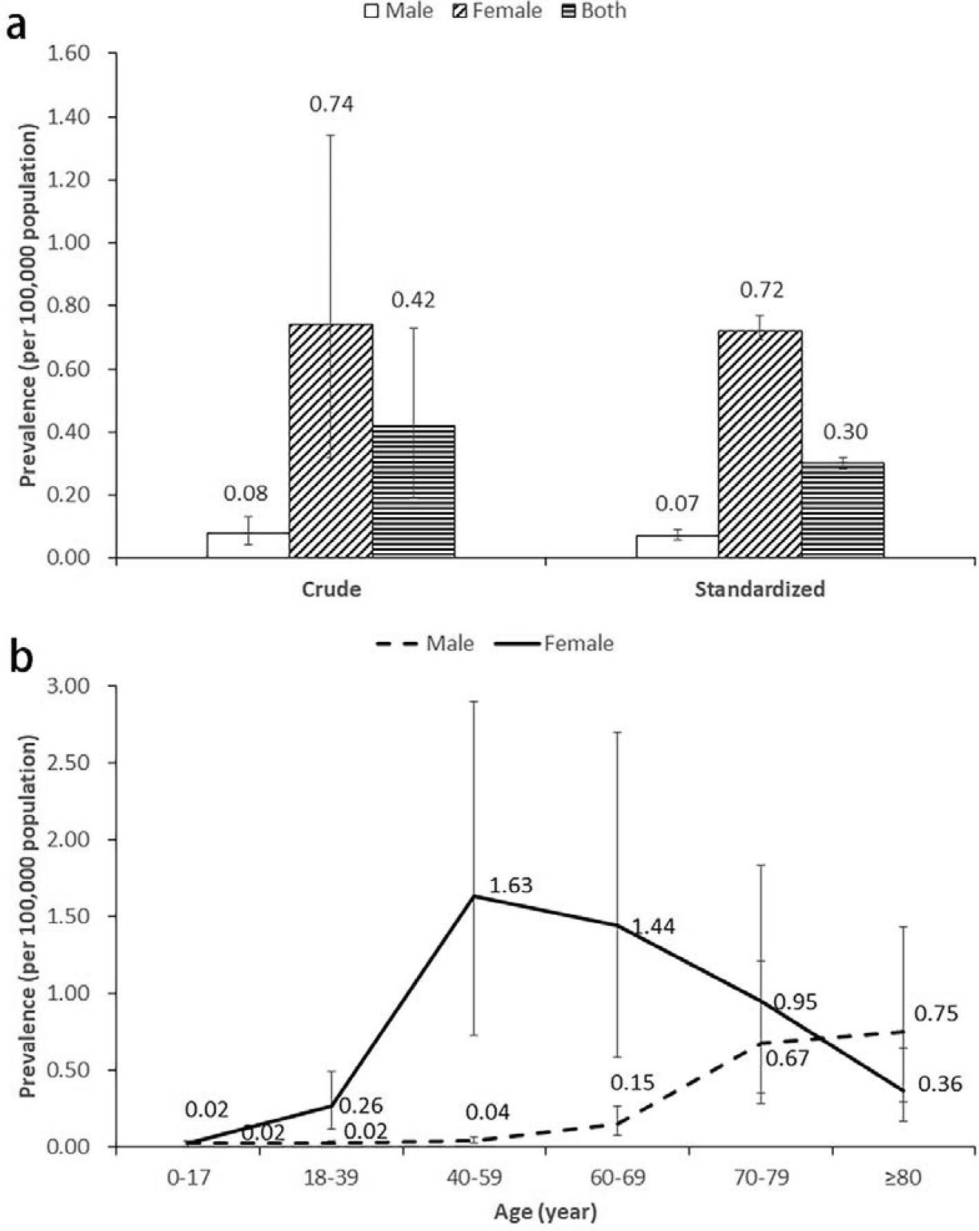
Prevalence of mammary Paget's disease in urban China in 2016. Note: the standardized prevalence is based on 2010 Chinese census data.

The prevalence of MPD in urban China in 2016 was 0.42 per 100,000 population (95% CI: 0.19 to 0.73) (Fig. 1). Based on the Chinese census data in 2016, there were 0.79 billion urban population, therefore in 2016, approximately 3,318 patients with MPD in China. The female prevalence (0.74, 95% CI 0.32 to 1.34) was higher than the male prevalence (0.08, 95% CI 0.04 to 0.13). As for the age trend of MPD prevalence, the prevalence peaked at 40–59 years old (1.63, 95% CI 0.72 to 2.90) among females, but among males, the prevalence kept rising with age, reaching the highest point at ≥ 80 years (0.75, 95% CI: 0.29 to 1.43). The MPD prevalence varied among different regions, ranging from 0.06 (95% CI 0.00 to 0.23) in Northeast China to 1.21 (95% CI 0.07 to 3.72) in Northwest China (Table 3).

**Table 3:** Unadjusted prevalence of mammary Paget's disease in urban population from 23 provinces in China in 2016 (unit: /100,000 population).

	Total (95% CI)	Male (95% CI)	Female (95% CI)
Total	0.42 (0.19 to 0.73)	0.08 (0.04 to 0.13)	0.74 (0.32 to 1.34)
*Age group*			
0–17	0.02 (0.01 to 0.03)	0.02 (0.00 to 0.03)	0.02 (0.01 to 0.04)
18–39	0.11 (0.05 to 0.19)	0.02 (0.01 to 0.04)	0.26 (0.11 to 0.49)
40–59	0.57 (0.26 to 1.00)	0.04 (0.02 to 0.06)	1.63 (0.72 to 2.90)
60–69	0.65 (0.32 to 1.09)	0.15 (0.07 to 0.26)	1.44 (0.58 to 2.70)
70–79	0.80 (0.44 to 1.27)	0.67 (0.28 to 1.21)	0.95 (0.35 to 1.83)
≥ 80	0.52 (0.29 to 0.83)	0.75 (0.29 to 1.43)	0.36 (0.16 to 0.64)
*Area*			
East	0.31 (0.06 to 0.76)	0.08 (0.04 to 0.12)	0.68 (0.05 to 2.08)
North	0.46 (0.02 to 2.23)	0.01 (0.00 to 0.03)	1.65 (0.00 to 6.74)
Northeast	0.06 (0.00 to 0.23)	0.01 (0.00 to 0.03)	0.17 (0.01 to 0.54)
Northwest	1.21 (0.07 to 3.72)	0.45 (0.00 to 1.68)	2.32 (0.02 to 10.16)
Southcentral	0.29 (0.02 to 0.87)	0.08 (0.01 to 0.19)	0.64 (0.00 to 2.58)
Southwest	0.10 (0.04 to 0.18)	0.05 (0.03 to 0.09)	0.15 (0.05 to 0.30)

### Age-adjusted prevalence

The age-adjusted national prevalence based on 2010 Chinese census data, the European, US and Australian populations were 0.30 per 100,000 population (95% CI 0.28 to 0.32), 0.36 (95% CI 0.34 to 0.38), 0.32 (95% CI 0.30 to 0.34) and 0.32 (95% CI 0.30 to 0.34), respectively. The age-adjusted prevalence rates of MPD were lower than the crude prevalence.

### Sensitivity analysis

The prevalence calculated by setting a stricter algorithm to identify target patients was 0.41 per 100,000 population (95% CI 0.19 to 0.73). By only considering the observed patients, we calculated the lower bound of the national prevalence as 0.20 (95% CI 0.09 to 0.35). Through excluding the top 10% of provinces with missing diagnostic information (i.e., Shandong and Xinjiang), the prevalence was 0.43 (95% CI 0.17 to 0.79).

## Discussion

In this population-based study of MPD, we elucidated three primary findings. First, MPD was significantly more prevalent in Chinese females, with a female to male ratio of around 9.4:1, and the prevalence in females was approximately nine times higher than that in males. This female predominance in our current study was consistent with previous studies^7,22,23^. A systematic review of published literatures using MEDLINE/PubMed and Google Scholar conducted in 2016 showed that only 24 cases of male MPD were identified in the published literatures since 1997^20^. In some previous studies, this discrepancy was directly attributed to the absolute predominance of breast cancer in females (male to female ratio 1:50–1:200)^2^. And the relatively better prognosis of female patients with MPD might also responsible for the higher prevalence in females^20,24^.

In our study, the prevalence of MPD in females was significantly lower than that in South Korea, which reported a prevalence of 0.091% between 1995 and 2019^25^. However, the hospital-based design in South Korea study could contribute to this evident discrepancy. The incidence rates of MPD were reported to range from 0.44 per 100,000 woman-years to 1.31 per 100,000 woman-years in the United States based on analysis of the SEER data between 1988 and 2011^9,26^. Considering the relatively good female prognosis of MPD^4,26,27^, the estimated prevalence in the United states during that period could be much higher than our current rate. Similar discrepancy also occurred in breast cancer studies. Although the population of China was approximately four times larger than that of the United States, the number of breast cancer cases among Chinese females per year was only 77% of that in the United States, and the prevalence in China was around one-fifth of that in the United States^28^. The ethnic difference between Chinese and American patients might be a possible explanation for the discrepancy. The overexpression of some essential factors in MPD, such as human epidermal growth factor receptor 2 (HER-2), was relatively lower in Chinese patients than that in the American patients^8^. And distributions in pathological characteristics of female breast cancer were also reported to be different between East Asian countries and the United States^29^. As for China, ductal carcinoma, lobular carcinoma and other types of breast cancer accounted for 78%, 4.99% and 17%, respectively^30^. It was reported that the five-year prevalence of breast cancer for Chinese females aged 15 years and older was 129 per 100,000 women^28^; therefore, the prevalences of ductal carcinoma, lobular carcinoma and other types of breast cancer were estimated to be 100.62 per 100,000 women, 6.44 per 100,000 women and 21.93 per 100,000 women. Given the MPD prevalence of 0.42 per 100,000 population we estimated, MPD is a very rare subtype of breast cancer; therefore, the statistics regarding MPD were not reported in the previous annual report, thus leading to the lack of basic epidemiological evidence of MPD, which further suggests the significance of this study.

Second, 62.68% of female patients were between 40 and 59 years old, with a mean age of 54.23. This was consistent with previous single- or multi-center hospital-based studies in mainland China^3,8,15^, but significantly younger than those of patients in the United States and Western Europe^9,23,26,31–34^. The relatively longer life expectancy in these developed western countries might have an impact on this difference^35^. However, racial disparity might also play a role, considering that patients with MPD in other Asian areas including the South Korea, Iran, Turkey, Saudi Arabia, Thailand and Taiwan of China were also much younger than those in Western populations^12–14,16–18,25,36^. A similar age discrepancy was observed in breast cancer between Chinese women and women from Western countries^28^. Previous studies have demonstrated the difference of tumour marker expression and tumour immune microenvironment between Asian and Western patients with breast cancer, which to some extent confirmed the existence of racial disparity^29,37^. However, detailed mechanisms concerning age discrepancy in patients with MPD is unknown and further studies are needed. Since the large proportion of female patients of MPD, the younger mean age of MPD patients in China compared with the United states and Western Europe implied that earlier mammographic screening for Chinese females is necessary^38^.

We also observed a clear difference between males and females for the patterns of MPD prevalence in China. The prevalence increased dramatically after the age of 39 in females, and decreased quickly after 60. By contrast, the rate in men increased with age, peaked after the eighth decade of life, and was more than twice the prevalence of females in the same age group. The exact reason for the discrepancy in China was unclear. In recent years, an increasing number of evidence suggested that male breast cancer might be a heterogeneous disease different from female breast cancer at molecular level^39^. The immunohistochemical profiles were significantly different between male and female breast cancers^40,41^. And some gene mutations found in female breast cancer, such as *BRIP1* and *RAD51C*, were not associated with male breast cancer^42–44^. These were all possible reasons for the differences in prevalence patterns between sexes. Moreover, a premenopausal female predominance was observed in Chinese patients with MPD^8^, which suggest a role of estrogen in the prevalence pattern of Chinese female patients. In addition, by taking Chinese medical policies into consideration, the following three points may explain this phenomenon as well. First, the Chinese government attaches great importance to maternal and child health, and China has a well-developed health care system for women and children, with medical insurance covering nearly all prefecture-level districts and counties^45^. Second, China implements the policy of annual physical examination among urban residents, and breast examination is included in it for women but not for men^46^. Also, the Two Cancer Screening Program (for screening breast cancer and cervical cancer among women)^47^ and National Urban Cancer Screening Program^48^ carried out in China further help improve the screening and detection rate of MPD for women but not for men. Third, annual mammograms for women between the ages of 40 and 60 were recommended in China's national guidelines since 2007^49^. Also, men's attitudes toward breast disease might also play a role to some extent. Compared with women, men’s awareness of breast disease and their active in medical treatment are generally lower, even if symptoms occur, patients are not willing to go to the hospital on time, which may lead to delayed diagnosis and relatively older patient group^50,51^. But the effects of males’ attitudes may be little and further studies were still required to explore the reasons for this phenomenon. The sex difference in age pattern of prevalence suggested that middle-aged females, the marjority of whom are in menopause, should pay more attention to the health status of their breasts, and require more detailed diagnosis when they suffer from eczematoid, erythematous, moist or crusted, itching lesions, and even accompanied by long-term recurrence of bleeding and skin ulcers of the breast and etc^52^, while for males especially those elder, it is also very important to keep an eye on the changes in their breast^53^, and doctors should also pay more attention to their breast during physical examination or surgical chest examination^54^.

Thirdly, the prevalence of MPD varied by the geographic areas of China, with the highest rate in Northwest China. It is noteworthy that Northwest China is the main dwelling area of ethnic minorities. A previous study in China have found different genotypes of *BRCA* germline mutation in breast cancer between multiple ethnicity region in Northwest China and ethnic Han Chinese in other regions, and suggested that this difference was caused by the interaction of genetic background and environmental factors^55^. But the relationship between *BRCA* germline mutation and MPD remained unclear, and information on mutations specifically predisposing MPD was also limited. Based on current data, it is difficult for us to determine the exact reason for the high MPD prevalence in Northwest China. However, to some extent, the influence of socioeconomic level and medical level on the prevalence of MPD could be excluded, as the Northwest China was relatively underdeveloped area in mainland China. The obvious regional disparities as the prevalence highest in Northwest China implies that the screening and control strategies regarding MPD should be adapted to local conditions^56,57^. Since the quality of medical service in economically disadvantaged Northwest China is lower, the local clinicians should be notified about MPD to fill the possible gaps in their knowledge, and further reduce missed diagnosis and misdiagnosis. Also, for better preventing and controlling MPD etiologically, carrying out etiological research in Northwest China to identify the potential risk factors is necessary.

We used a large, nationally representative sample of the Chinese urban population which ensured the overall estimation of the prevalence of a rare disease like MPD. This nationally representative data also enabled us to explore age and sex patterns of the prevalence as well as regional differences in China. This study also has several limitations. First, the basic medical insurance database lacks some detailed information, such as tumour stage, clinical characteristics, results of pathological examination and laboratory results, which limits the possibility to stratify the diagnosis in greater detail and analyze the data in more depth, similar to other previous studies based on claim data^58,59^. Second, rural inhabitants and certain urban populations, such as military soldiers are not included in the UEBMI and URBMI system because they have different types of medical insurance. The exclusion of these groups could have affected the estimates.

This population-based study investigated the prevalence of MPD in urban China based on the basic medical insurance database. The prevalence of MPD showed a significant female predominance in China, and the rate in females was much lower than that in the United States. Female patients with MPD in China was much younger than those in developed western countries. In males, MPD were more prevalent at an older age after 80. These findings add to our understanding of the epidemiologic characteristics of MPD in China, and at the same time, provide important implications for further epidemiological studies of MPD worldwide.

## Materials and methods

### National medical insurance database

Two main medical insurance schemes for Chinese urban population are Urban Employee Basic Medical Insurance (UEBMI) and Urban Residence Basic Medical Insurance (URBMI). UEBMI is for employees no matter working or retired (i.e., employers and employees in government agencies and institutions, social organizations, state enterprises, as well as other private businesses. URBMI is for unemployed urban citizens (i.e., children, students, the elderly, etc.) The data in UEBMI and URBMI is updated at the city level monthly. No matter the proportion the insured people paid for the medical service, their reimbursement records will be kept in the database. Information including the sociodemographic characteristics (i.e., ethnicity, sex, birth date, place of residence, etc.), diagnostic information (i.e., disease names, disease codes, etc.), as well as medical expenses can be found in the database. The two medical insurance schemes covered more than 95% of Chinese urban population in 2016^60,61^. The detailed introduction of the two medical insurance schemes can be found in previous studies^62–64^. This study is registered with the Chinese Clinical Trial Registry (ChiCTR), number ChiCTR1800018217.

### Study population

UEBMI and URBMI data of 23 provinces between January 1st, 2016 and December 31st, 2016 were used to conduct this retrospective population-based study. Consistent with our previous work^64,65^, we excluded the data of eight provinces due to absence or abnormality of crucial information such as diagnostic information (Beijing, Shanghai, Sichuan, Ningxia, Hebei), only containing one insurance type (Tianjin), reporting policy exemptions (Fujian and Tibet). We kept all claim records de-identified to protect subjects’ privacy. The study protocol was approved by the ethical review committee of the Peking University Health Science Center (IRB. No.: IRB00001052-18012), and the informed consent requirement was waived.

### Case identification

Target patients were identified using the diagnostic information in the database (i.e., the diagnostic text and International Classification of Diseases (ICD) code). We applied the natural language processing to normalize the diagnostic information with a dictionary of potential MPD defined by prestigious clinicians. Potential patients with MPD were selected by ICD-10 (M85400/3, M85400/6, C50.903, M85410/3, C50.904, M85410/6, M85430/3, M85430/6) and Chinese medical terms of diseases including MPD, mammary eczematoid carcinoma. Diagnostic information of each potential target patient was then judged by two prestigious clinicians independently to identify actual patients with MPD. Any disagreements between them were solved by another senior clinician. The detailed case identification process can be seen in the Supplementary file. Since the reimbursement records of the insured population will be kept in the databases if they provide the national insurance card for the medical service (even though no medical expense was reimbursed), almost all patients with MPD in 2016 data can be identified.

### Statistical analysis

To calculate the national prevalence of MPD in 2016, we adopted a two-stage approach, which was described in detail in our previous studies for calculating the prevalence of other rare diseases^64,65^. In the first stage, we calculate the prevalence of MPD in each province. In the second stage, the national or regional prevalence was calculated by pooling the prevalence of each province via random effects meta-analysis. To stabilize the variance of province-level prevalence, the Freeman-Tukey double arcsine transformation was used.

Based on the 2010 Chinese national census data, the Revised European Standard Population (RESP) 2013, the 2010 US population and the 2011 Australian population, we calculated the age-adjusted prevalence rates. We also performed subgroup analyses by sex, age, and geographical area (East, North, Northeast, Northwest, Southcentral, and Southwest). We used sensitivity analyses to assess the robustness of the main results by setting a stricter algorithm (not considering the patients with diagnostic information containing eczematoid carcinoma) to identify actual target patients, by only considering the observed target patients to estimate the lower bound of the prevalence, and by excluding the top 10% of provinces with missing diagnostic information. Poisson distribution was used to estimate the 95% CIs of prevalence. We used Student's t-test for continuous variables and the chi-squared test for categorical variables. All statistical analyses were done by Stata 15.0 (StataCorp, College Station, TX, USA), and a two-sided *P* < 0.05 was considered as statistically significant.

## Ethical statement

The study protocol was approved by the ethical review committee of the Peking University Health Science Center (IRB. No.: IRB00001052-18012), and the informed consent requirement was waived. All methods were carried out in accordance with the Declaration of Helsinki.

## Supplementary Information

Supplementary Information.

## Data Availability

The data that support the findings of this study are available on request from the corresponding author. The data are not publicly available due to privacy or ethical restrictions.
