# Supplementary material for: Prevalence of mammary Paget’s disease in urban China in 2016
Source: Sci Rep. 2021 Jan 28;11:2572. doi: 10.1038/s41598-021-82146-y (PMC7844003; doi:10.1038/s41598-021-82146-y)
Supplement: Supplementary file 1 — Supplementary Information. [file 41598_2021_82146_MOESM1_ESM.docx]

**Title:** Prevalence of Mammary Paget’s Disease in Urban China in 2016

**Authors:** Lu Xu^1#^ PhD candidate, Shilu Yin^2#^ MD, Shengfeng Wang^1*^ PhD, Jingnan Feng^1^ MMsc candidate, Lili Liu^1^ MMsc candidate, Guozhen Liu^3^ BS, Jinxi Wang^4^ BS, Siyan Zhan^1, 5, 6*^ PhD, Zhenmin Zhao^2*^ MD, Pei Gao^1*^ MD

**Author affiliations**:

1 Department of Epidemiology and Biostatistics, School of Public Health, Peking University, 38 Xueyuan Road, Haidian District, Beijing 100191, China

2 Department of Plastic Surgery, Peking University Third Hospital, 49 North Garden Road, Haidian District, Beijing 100191, China

3 Peking University Health Information Technology Co. Ltd, 52 North Fourth Ring West Road, Haidian District, Beijing 100080, China

4 Shanghai Songsheng Business Consulting Co. Ltd, 6 Chaoyang Men North street, Dongcheng District, Beijing 100000, China

5 Research Center of Clinical Epidemiology, Peking University Third Hospital, 49 North Garden Road, Haidian District, Beijing 100191, China

6 Center for Intelligent Public Health, Institute for Artificial Intelligence, Peking University, 38 Xueyuan Road, Haidian District, Beijing 100191, China

^#^ Lu Xu and Shilu Yin are joint first authors.

**Corresponding authors:**

Shengfeng Wang PhD, affiliation: Department of Epidemiology and Biostatistics, School of Public Health, Peking University, e-mail: shengfeng1984@126.com, telephone: 86-10-82801528, mailing address: 38 Xueyuan Road, Haidian District, Beijing 100191, China;

Siyan Zhan PhD, affiliation: Department of Epidemiology and Biostatistics, School of Public Health, Peking University, e-mail: siyan-zhan@bjmu.edu.cn, telephone: 86-10-82805162, fax: 86-10-82805162, mailing address: 38 Xueyuan Road, Haidian District, Beijing 100191, China;

Zhenmin Zhao MD, affiliation: Department of Plastic Surgery, Peking University Third Hospital, e-mail: zzmbysy@sina.com, telephone: 86-10-82267340, mailing address: 49 North Garden Road, Haidian District, Beijing 100191, China

Pei Gao MD, affiliation: Department of Epidemiology and Biostatistics, School of Public Health, Peking University, e-mail: peigao@bjmu.edu.cn, telephone: 86-10-82805642, fax: 86-10-82805642, mailing address: 38 Xueyuan Road, Haidian District, Beijing 100191, China

**Supplementary materials**

**The case identification process**

First, texts containing Paget’s disease diagnostic information were extracted from the medical insurance databases. To avoid missing patients when using the medical terms in Chinese, a fuzzy string-matching algorithm was used to extract potential Paget’s disease patients from the databases. Keywords were first defined according to ICD-10 codes, tumour morphologic codes and medical terms in Chinese (i.e., different Chinese transliteration names of Paget), shown in **Supplementary Table S1**. The search algorithm was therefore defined as:

**(C51.9 OR M85400/3 OR C50.9).* OR .*( Paget病 OR Paget′s病 OR Paget氏病) .* OR .* (帕哲病 OR 帕哲氏病 OR 派杰病 OR 派杰氏病 OR 佩吉特病 OR 佩吉特氏病 OR 派吉病 OR 派吉氏病 OR 佩吉病 OR 佩吉氏病 OR 柏哲德病 OR 柏哲德氏病) .* OR .* (湿疹样癌 OR 皮肤湿疹样癌 OR 湿疹样病变 OR 皮肤湿疹样病变)*.

**Supplementary Table S1 Keywords used for extracting potential Paget’s disease cases**

| **Diagnostic name** | **Chinese characters** | **ICD-10** | **Tumour morphologic codes** |
| --- | --- | --- | --- |
| **Mammary Paget’s disease** | | | |
| Mammary Paget’s disease | 乳房佩吉特病 | C50.903 | M85400/3 |
| Mammary Paget’s disease | 乳房佩吉特氏病 | C50.903 | M85400/3 |
| Mammary Paget’s disease | 乳房Paget病 | C50.903 | M85400/3 |
| Mammary Paget’s disease | 乳房Paget’s病 | C50.903 | M85400/3 |
| Mammary Paget’s disease | 乳房Paget氏病 | C50.903 | M85400/3 |
| Mammary Paget’s disease | 乳房帕哲病 | C50.903 | M85400/3 |
| Mammary Paget’s disease | 乳房帕哲氏病 | C50.903 | M85400/3 |
| Mammary Paget’s disease | 乳房派杰病 | C50.903 | M85400/3 |
| Mammary Paget’s disease | 乳房派杰氏病 | C50.903 | M85400/3 |
| Mammary Paget’s disease | 乳房派吉氏病 | C50.903 | M85400/3 |
| Mammary Paget’s disease | 乳房派吉病 | C50.903 | M85400/3 |
| Mammary Paget’s disease | 乳房柏哲德氏病 | C50.903 | M85400/3 |
| Mammary Paget’s disease | 乳房柏哲德病 | C50.903 | M85400/3 |
| Mammary eczematoid carcinoma | 乳房湿疹样癌 | C50.903 | M85400/3 |
| Mammary Paget’s disease with invasive ductal carcinoma | 乳房佩吉特氏病伴浸润性导管癌 | C50.904 | M85410/3 |
| Metastatic mammary Paget’s disease and invasive ductal carcinoma | 转移性乳房佩吉特病和浸润性导管癌 | - | M85410/6 |
| Mammary Paget’s disease and intraductal carcinoma | 乳房的佩吉特病和导管内癌 | - | M85430/3 |
| Metastatic mammary Paget’s disease and intraductal carcinoma | 转移性乳房佩吉特病和导管内癌 | - | M85430/6 |
| Metastatic mammary Paget’s disease | 转移性乳房佩吉特病 | - | M85400/6 |
| **Extramammary Paget’s disease** | | | |
| Extramammary Paget’s disease | 乳房外佩吉特病 | C50.903 | M85400/3 |
| Extramammary eczematoid carcinoma | 乳房外湿疹样癌 | C50.903 | M85400/3 |
| Extramammary Paget’s disease of vulval | 外阴佩吉特病 | C51.902 | - |
| Extramammary Paget’s disease of scrotum | 阴囊佩吉特病 | C63.252 | M85400/3 |
| Extramammary Paget’s disease (except for paget's disease of the bone) | 乳房外佩吉特病（除外骨的佩吉特病） | - | M85420/3 |
| Metastatic extramammary Paget’s disease | 转移性乳房外佩吉特病 | - | M85420/6 |

Second, based on the searching algorithm, two prestigious clinicians performed manual verification of the diagnostic text with the assistance of the computer algorithm. The flow-chart for the case ascertainment was shown in **Supplementary Fig. S1**. The inclusion criteria for patients with Paget’s disease were defined as patients with (1) Paget’s disease; (2) eczematoid carcinoma; (3) ICD-10 codes (C50.903, C50.904, C51.902, C63.252); (4) Tumour morphologic codes (M85400/3, M85400/6, M85410/3, M85410/6, M85430/3, M85430/6, M85420/3, M85420/6). If the diagnostic text contained words such as “undetermined”, “uncertainty”, “?”, and other similar synonyms, these patients were categorized as a subgroup named “patients with uncertainty of Paget’s disease”, and these patients were excluded.

As shown in **Supplementary Fig. S1**, in the verification stage, first of all, we generated the codebook for tagging Paget’s disease. Total 7,036 unique diagnostic expression combination (i.e., six variables of diagnostic text or code were combined and separated by commas) were obtained using the searching algorithms defined above from both UEBMI and URBMI. These expressions were then segmented using Jieba Chinese word segmentation tool to gain unique diagnostic terms, according to a clinical terminology database. Overall, 4,917 terms were obtained. Then, a cosine distance function, shown as **Supplementary Equation S1**, was defined to measure the similarity between the diagnostic term and standard definition for Paget’s disease case (**Supplementary Fig. S1**).

$$\sin\left( X,Y \right)=\cos\theta=\frac{\vec{x}\cdot\vec{y}}{\left\| x \right\|\cdot\left\| y \right\|} (S1)$$

where X and Y represented the vector of query and matching word respectively.

The terms with cosine similarity ≥ 0.6 or containing the standard diagnostic terms of Paget’s disease or eczematoid carcinoma were considered as potential Paget’s disease related terms, and then, these terms were reviewed by two prestigious clinicians independently to create a codebook of Paget’s disease related terms for tagging.

Third, we used the tagging codebook to classify the records from both UEBMI and URBMI based on the searching algorithm, i.e. individual records were categorized into those with or without tagging text. For records with tagging text, two prestigious clinicians reviewed them independently to further exclude the inappropriate expression, and classify the Paget’s disease into mammary and extramammary Paget’s disease according to the criteria shown in **Supplementary Table S1**. For records without tagging text, the cosine distances of the diagnostic terms from these records were calculated. Two thresholds, i.e. 0.4 and 0.6 were used to classify individual terms. If the cosine similarity of all the diagnostic terms in a record was less than 0.4, the record was discarded. If the highest cosine similarity in a record was between 0.4 and 0.6, the record was reviewed by two prestigious clinicians independently to further include the potential missing target patients. If we find any, the related records were tagged. In our study, no records with the highest cosine similarity between 0.4 and 0.6 were judged as Paget’s disease by prestigious clinicians. Finally, 8,149 records from 825 patients with MPD were included in our study.


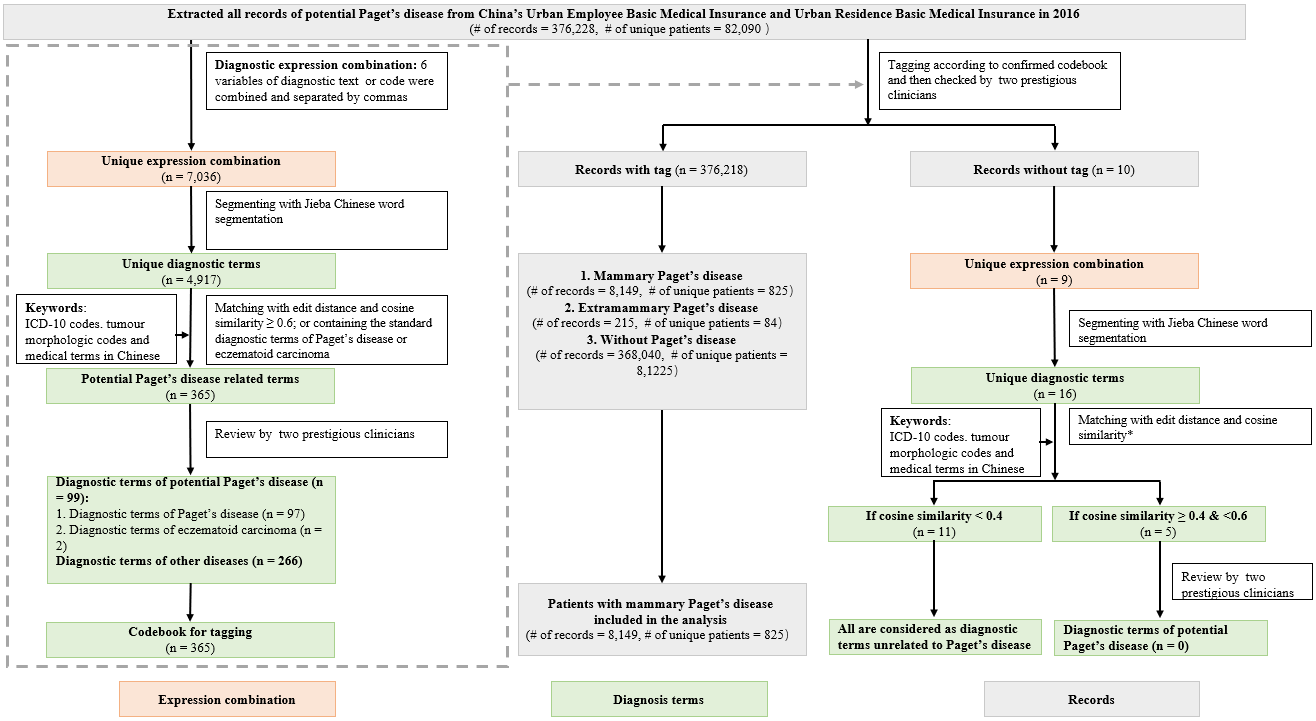


**Supplementary Fig. S1 Flow chart of case ascertainment.**

Note: If the cosine similarity of all terms in a record was less than 0.4, the record was discarded. If the cosine similarity was between 0.4 and 0.6, the record was reviewed by two prestigious clinicians independently to further include the potential missing patients with mammary Paget’s disease.
